# The CFIR Card Game: a new approach for working with implementation teams to identify challenges and strategies

**DOI:** 10.1186/s43058-020-00099-1

**Published:** 2021-01-07

**Authors:** Myra Piat, Megan Wainwright, Eleni Sofouli, Hélène Albert, Regina Casey, Marie-Pier Rivest, Catherine Briand, Sarah Kasdorf, Lise Labonté, Sébastien LeBlanc, Joseph J. O’Rourke

**Affiliations:** 1 Department of Psychiatry, McGill University, Douglas Hospital Research Centre, Montreal, Quebec Canada; 2 Department of Anthropology, Durham University, Durham, UK; 3 École de Travail Social, Université de Moncton, Moncton, New Brunswick Canada; 4 Department of Psychology, Douglas College, New Westminster, British Columbia Canada; 5 Department of Occupational Therapy, Université du Québec à Trois-Rivières, Trois-Rivières, Québec Canada; 6 Department of Occupational Science and Occupational Therapy, University of British Columbia, Vancouver, British Columbia Canada

**Keywords:** Consolidated Framework for Implementation Research, Expert Recommendations for Implementing Change, Games, Implementation teams, CFIR-ERIC Matching Tool, Mental health recovery, Housing, Guidelines, Canada

## Abstract

**Background:**

The Consolidated Framework for Implementation Research (CFIR) and the ERIC compilation of implementation strategies are key resources for identifying implementation barriers and strategies. However, their respective density and complexity make their application to implementation planning outside of academia challenging. We developed the CFIR Card Game as a way of working with multi-stakeholder implementation teams that were implementing mental health recovery into their services, to identify barriers and strategies to overcome them. The aim of this descriptive evaluation is to describe how the game was prepared, played, used and received by teams and researchers and their perception of the clarity of the CFIR constructs.

**Methods:**

We used the new CFIR-ERIC Matching Tool v.1 to design the game. We produced a deck of cards with each of the CFIR-ERIC Matching Tool barrier narratives representing all 39 CFIR constructs. Teams played the game at the pre-implementation stage at a time when they were actively engaged in a planning process for implementing their selected recovery-oriented innovation. The teams placed each card in either the YES or NO column of the board in response to whether they anticipated experiencing this barrier in their setting. Teams were also asked about the clarity of the barrier narratives and were provided with plain language versions if unclear. Researchers completed a reflection form following the game, and participants completed an open-added questionnaire that included questions specific to the CFIR Card Game. We applied a descriptive coding approach to analysis.

**Results:**

Four descriptive themes emerged from this analysis: (1) the CFIR Card Game as a useful and engaging process, (2) difficulties understanding CFIR construct barrier narratives, (3) strengths of the game’s design and structure and room for improvement and (4) mediating factors: facilitator preparation and multi-stakeholder dynamics. Quantitative findings regarding the clarity of the barrier narratives were integrated with qualitative data under theme 2. Only seven of the 39 original barrier narratives were judged to be clear by all teams.

**Conclusions:**

The CFIR Card Game can be used to enhance implementation planning. Plain language versions of CFIR construct barrier narratives are needed. Our plain language versions require further testing and refining.

**Supplementary Information:**

The online version contains supplementary material available at 10.1186/s43058-020-00099-1.

Contributions to the literature

- With the existence of robust compilations of barriers and facilitators (CFIR) and strategies (ERIC), and new tools like the CFIR-ERIC Matching Tool v.1 to connect the two, our work has turned to putting these into practice with implementation teams.
- Our CFIR Card Game enhances implementation planning by helping implementation teams identify potential barriers early and match these to strategies for integration into their plans.
- The game offers a process for both bringing together known implementation strategies (establishing implementation teams and assessing for barriers and facilitators) and for using the new CFIR-ERIC Matching Tool v.1.

## Background

The consolidated framework for implementation research (CFIR) is a compilation of factors known to affect implementation which are categorized into five domains: intervention characteristics, outer setting, inner setting, characteristics of individuals and process [1]. For implementation science researchers and academics, it offers a framework and shared vocabulary for developing implementation-relevant research questions, data collection tools and approaches to analysis [2]. CFIR’s relevance however goes beyond academic research. In practice, it is a powerful tool for decision makers and change makers in organizations to plan for implementation of new innovations into their settings. A related step in the planning process is to consider implementation strategies, that is methods or techniques that can be employed to help overcome or minimize the impact of barriers, thus enhancing implementation success. A compilation of 73 implementation strategies and their definitions was produced by the Expert Recommendations for Implementing Change (ERIC) project [3]. With the existence of these two robust resources, one for identifying implementation challenges (CFIR) and another for strategies to overcome them (ERIC), the focus has turned to building an evidence base for linking the two—which strategies best address which barriers?

The CFIR-ERIC Matching Tool v.1 is a new, freely available tool for helping researchers match individual barriers to expert-endorsed implementation strategies [4]. The tool was developed by asking 169 implementation experts to select and rank up to seven strategies from the list of ERIC strategies that would best address each CFIR construct. For this purpose, CFIR constructs were written-up as barrier narratives (one sentence statements) to illustrate the meaning of the CFIR construct. For example, the barrier narrative for the CFIR construct “Adaptability” is “Stakeholders do not believe that the innovation can be sufficiently adapted, tailored, or re-invented to meet local needs.” (Additional file 1).

Users of the CFIR-ERIC Matching Tool v.1 select the barriers they face and at the click of a button, a matrix table is generated in a separate excel tab with all the selected barriers listed in the first row and all the 73 strategies listed in the first column. The cell at the intersection of any given row and column shows the percentage of surveyed experts who selected this strategy as one of their top seven strategies for this barrier. A level 1 endorsement is a strategy for which 50% or more of the experts surveyed said it was a top seven strategy. Level 2 endorsements are those for which between 20 and 49.9% of experts surveyed put that strategy in their top seven for that barrier. The authors conclude that “because of the wide diversity of responses by our expert respondents and the lack of consensus this represents for the majority of endorsements, this tool must be used with caution” (p.6) but that “The CFIR-ERIC Mapping Tool could be used to generate a list of ERIC strategies to consider for addressing each CFIR barrier” (p.9) [4].

We drew on the CFIR-ERIC Matching Tool v.1 to transform the CFIR into the CFIR Card Game to be played with implementation teams across Canada. This was carried out within the research project—*Implementing Mental Health Recovery Guidelines into Services: A Pan Canadian Study* [5]. The aim of the research project was to translate the Canadian Mental Health Commission Guidelines for Recovery-Oriented Practice [6] into tangible innovations to be implemented into seven organizations providing services, primarily housing, to adults with mental health problems, across five Canadian provinces. Recovery-oriented services are those that espouse the values of person-orientation, person involvement, self-determination/choice and hope [7]. Services that are recovery-oriented support personally-defined recovery, that is support people living with serious mental illness “to define their own needs, goals, dreams, and plans for the future” (p.1474) [8] with a focus on connectedness, hope and optimism about the future, identity, meaning in life and empowerment [8].

The overall strategy for optimizing acceptability, feasibility and overall buy-in was to not pre-select an evidence-based intervention to be implemented across all sites, but rather establish an implementation team at each site “charged with designing and implementing an organization-wide change strategy” (p.382) [9]. Implementation teams are themselves a powerful implementation strategy [3] and considered a critical step for quality implementation [10]. Implementation teams included tenants or service users, service providers, family members, managers and knowledge users, achieving “positional diversity” on the team (p.371) [9]. Knowledge users were defined as individuals external to the organization with the ability to use the produced knowledge to inform policies and programs [11]. Service users play an important role in implementation [12] and their inclusion alongside family members on implementation teams was part of our recovery-oriented approach to the research as a whole [13].

A research team at each site operated as external change agents [1] in the CFIR sense of the word—instigating, guiding and supporting the change process. Over the course of 6 months (between November 2018 and July 2019), research teams in each site worked intensively with implementation teams through 12 implementation team meetings (lasting 2 h every 2 weeks). The 12 meetings were methodically planned by the national-level research team (MP, MW, ES) and guided the teams towards completing an Action Plan consisting of 6 milestones which were (1) selecting a sub-guideline, (2) defining the innovation (three sites chose Recovery Training, two sites Hiring Peer Workers, one site Wellness Recovery Action Planning and one site Family Support Groups), (3) anticipating facilitators and barriers, (4) defining implementation strategies, (5) engaging stakeholders and (6) writing an implementation plan. Four sites were led by the principal investigator (MP) and each of the remaining three sites by one of the co-investigators (M-PR, HA, RC). Research assistants (ES, JO, SL, SK, LL) co-facilitated each of the 12 meetings, with three working at more than one site (ES, SL, SK). The CFIR Card Game was played over meetings 8 and 9 to achieve the milestone of anticipating facilitators and barriers (milestone 3). The results of the game were used in meeting 10 to achieve the milestone of defining implementation strategies (milestone 4). We drew on interactive, game-based approaches to enliven meetings and make the content more accessible. Games can reduce anxiety by increasing enjoyment of the learning process, particularly in situations where learners’ confidence is low and anxiety is high [14]. We believe games are a promising way of approaching knowledge translation [15] of complex implementation theory and frameworks to multi-stakeholder implementation teams. The detailed process of establishing teams and the content for each meeting as well as the Action Plan and milestones is the subject of a separate publication (Piat et al.: Translating mental health recovery guidelines into recovery-oriented innovations: A strategy combining implementation teams and a facilitated planning process, under review).

We designed the game to take into consideration some key findings of Kirk et al.’s [11] review of the use of CFIR [2]. Firstly, researchers rarely justified their choice of domains and constructs to focus on. Secondly, CFIR has most often been used at the post-implementation stage rather than during earlier phases. Thirdly, research has tended to evaluate barriers and facilitators at one point in time. Fourthly, researchers employing CFIR have generally used it *on* participants (as a data collection tool) rather than *with* participants as an implementation planning process itself. We designed the game so that implementation teams could identify for themselves the constructs to focus on at the pre-implementation stage. Although we did play the game a second time with teams for them to re-evaluate barriers and strategies at the implementation stage, the current manuscript focuses only on the first iteration of the game at the pre-implementation planning stage. Overall, the game provided a more approachable way of introducing teams to a large number of constructs (39 in total). The use of the CFIR-ERIC Matching Tool v1 to match these to strategies facilitated the process of using the ERIC compilation of strategies by generating a smaller, more manageable list of targeted strategies matched to challenges.

Implementation teams and the identification of barriers and facilitators are well-known implementation strategies [9]. The CFIR Card Game brings together these implementation strategies through a novel process that can be used to enhance implementation planning. The aim of this descriptive evaluation is to describe how the game was prepared, played, used and received by teams and researchers and their perception of the clarity of the CFIR construct barrier narratives [4] used as the basis of the game. We applied the Standards for Reporting Implementation Studies (StaRI) checklist as a reporting guide [16].

## Methods

### Preparing the CFIR Card Game

Each of the 39 barrier narratives from the CFIR-ERIC Matching Tool v.1 were printed on individual cards divided into the five CFIR domains. A poster board was created for each domain with the question “Is this going to be a challenge/hurdle when implementing our innovation?” at the top and a YES and NO column below. We opted for the words challenge and hurdle rather than barrier as we felt barrier connoted a certain fatalism and negativity that would strike the wrong chord with participants. In all meetings and during the game, we referred to the barrier narratives as “the challenges” as we will in this article. Materials were prepared in both French and English for sites in Québec and New Brunswick.

Clarifying language when it comes to translating implementation science concepts is an important part of putting implementation science into action [17]. Taking into consideration this call for clarifying concepts, we reflected on the language used in the barrier narratives and anticipated that the vocabulary and phrasing might be difficult for non-academic audiences to understand. Therefore, three researchers (MP, MW, ES) worked together to draft slightly modified versions (we refer to these as the plain language versions) of the barrier narratives that used, in our opinion, simpler and more direct language where possible (see original barrier narratives [4] and our plain language versions in Additional file 1). To not assume that our perception that some were hard to understand was correct, we decided that the originals would be used in the game but if the team found any unclear we would read our alternative plain language version.

To provide some overall context for implementation science and the CFIR, we selected a short video introducing implementation science to play before starting the game [18]. We also prepared a PowerPoint presentation to introduce the five CFIR domains which we described in more descriptive terms (e.g. outer setting = the world outside the organization; inner setting = the organization; intervention characteristics = the innovation; characteristics of individuals = the people involved and process = the plan). The presentation also included the instructions for the game and each of the barrier narratives that would appear on the cards. All researchers who facilitated the game were provided with a detailed facilitator’s guide which was introduced and explained in a research team meeting. Additional coaching from the coordinator was available upon request.

In terms of the structure of the game, the following minor changes were made to the process (but not the content) after we played the game in the first site. We changed the order and timing of the rounds from outer setting and inner setting at meeting 6 and the other three at meeting 7, to inner setting in the second half of meeting 8 (since this is the domain with the most constructs) and intervention characteristics, outer setting, characteristics of individuals and process during the whole of meeting 9 (approximately 2.5 to 3 h in total). These choices were made for logistical reasons and were not based on assumptions of what order the domains would best be played in.

### Playing the CFIR Card Game

The game was divided into five rounds, one for each CFIR domain. The game was facilitated by one researcher while a second researcher observed and took notes. Implementation team members were asked to, each in turn, pick a card from the deck in the centre of the table and read it out loud to the group. The corresponding text was simultaneously projected so that participants could both hear and read the cards. The card was read and teams were asked “is it clear”? If the resounding answer from the group was no, the plain language version was projected and read. Then, the group was asked whether they would answer YES or NO to the following question regarding the statement on the card they just heard/read “Is this going to be a challenge/hurdle when implementing our innovation?” The answer was reached through consensus, but the game was fast-paced, and so, if after 1 minute no consensus was obvious, the facilitator took a vote through show of hands. The facilitator prompted for reasons and explanations. The person who read the card placed it in the corresponding column on the poster board and the next player picked a card.

All the cards placed in the YES column were taken as challenges implementation teams identified for their setting. Researchers were advised to reassure teams that challenges were normal and by identifying them they would be able to plan strategies to help overcome them. In addition to being audio-recorded, the co-researcher observing filled in a CFIR Game Recording Sheet to record answers and note any observations (e.g. silences, disagreements). Photographs were also taken of the poster boards as a record of the team’s responses.

### Using the results of the game

The results were presented in the section Anticipating Facilitators and Barriers (milestone 3) of the Action Plan which was printed out and distributed to team members at meeting 10. This gave teams access to the full list of challenges they identified which they could return to at any time in the planning process. The research team entered their responses from the game into the CFIR-ERIC Matching Tool v.1, which generated a matrix of the most to least expert-endorsed strategies to address each identified barrier. Our challenge was to decide how to use the results of the tool. We reached out to the CFIR-ERIC Matching Tool v.1 [4] developers for some clarification as we considered two options: (1) provide teams with a package of the ten strategies with the greatest cumulative percentages (strategies that were identified as being top 7 strategies for more than one of the challenges identified) or (2) individually map each identified challenge to the highest-endorsed strategies. While both had merits, we chose the latter as we thought it would be clearer to teams to see strategies for each challenge they identified. The research team populated the first part of the Defining Implementation Strategies section of the Action Plan (milestone 4) with each challenge identified in the first column and corresponding level 1 endorsement strategies (meaning those with highest expert endorsement) in the second column. If there were no level 1 strategies for a barrier, then we selected the highest level 2 strategies. If there was more than one strategy with the same percentage, all were listed. This ensured that the list of strategies matched to each of their identified barriers were at the team’s disposal and became part of the Action Plan they could return to throughout the implementation process.

To help the teams learn how to plan the use of a strategy, the researchers facilitated the process proposed Proctor et al. [19] for defining and specifying strategies at meeting 10. Three strategies were pre-selected for the exercise by the researchers in each site based on their contextual knowledge of their site. The fact that the barriers were identified by teams themselves and that the CFIR-ERIC Matching Tool v.1 helped reduce the starting list of strategies, strongly facilitated the process of choosing three strategies to prioritize at meeting 10. The reason for researchers’ selecting the strategies was purely pragmatic. If we had two meetings devoted to strategies, we would have built-in a consensus-building process, but our experience had taught us that we were unlikely to be able to do both (collaboratively choose strategies and apply the process for defining and specifying) in one meeting. By using the game and the CFIR-ERIC Matching Tool, prioritization of three strategies to discuss in the meeting was not based on researchers’ informed choice alone, but also on implementation experts’ experiences and perceptions of which strategies work best for which barriers. The idea was that the teams would be taught a process in meeting 10 which they could then apply to any of the other strategies that had been matched to their identified barriers (milestone 4). The three strategies were a starting point, rather than an endpoint. Researchers were asked to consider prioritizing strategies that were matched to multiple challenges in the CFIR-ERIC Matching Tool v.1. They were also asked to keep in mind that engaging stakeholders and writing an implementation plan were strategies we would explicitly address in meetings 11 and 12. Strategies chosen were as follows: “Identify and Prepare Champions” (all teams), “Accessing New Funding” (four teams), “Involve Consumers and Family Members” (three teams), “Conduct Educational Meetings” (two teams), “Organize Stakeholder Implementation Team Meetings” (one team) and “Promote Adaptability” (one team).

### Evaluating the game—data collection

Table 1 shows the number of implementation team members in each site who played the game. The CFIR Card Game Recording Sheets were completed in each site to record whether the group responded “yes” or “no” to the question is it [the barrier narrative] clear? A group response of “no” was recorded if that was the consensus position of the group (individual level data was not collected). Qualitative data specific to the CFIR Card Game was collected via researcher reflection forms that included questions targeting researchers’ reflections on understanding, transfer of knowledge, participation, group dynamics and their perception of how well the game went. Twelve researcher reflection forms were completed in all, representing at least one report per site. At the end of the 12-meeting process, all implementation team members were invited to complete an open-ended questionnaire collecting qualitative data for three questions regarding the CFIR Card Game (What did you like about it? What did you not like about it? Do you have suggestions for improving it?). Table 2 reports characteristics of the team members who completed the open-ended questionnaire. Of the 54 team members who completed the open-ended questionnaire, 47 responded to the CFIR Card Game questions (Table 1) and seven (3 service users, 2 service providers, 1 knowledge user and 1 family member) left the section blank or indicated they did not remember the game.

**Table 1:** Number of implementation team members who played the CFIR Card Game

Implementation team (site)	Number of team members at meeting 8	Number of team members at meeting 9
Québec	11	7
Ontario	5	8
Manitoba 1	9	7
Manitoba 2	5	7
New Brunswick 1	6	5
New Brunswick 2	7	7
British Columbia	9	7
Totals	**52**	**49**

**Table 2:** Demographic characteristics of members of the implementation teams who completed the open-ended questionnaire

Stakeholder group	Sample size (***N*** = 54)	Gender	Age in years (M, SD)
Service users	*n* = 16	8 female/7 male 1 missing value	52 (13.5)
Family members	*n* = 3	3 female	50.67 (17.7)
Service providers	*n* = 19	11 female/7 male/1 non-binary	42.57 (14.42)
Managers	*n* = 12	10 female/2 male	44.9 (11.63)
Knowledge users	*n* = 4	3 female/2 male	44.6 (9.04)

*M* means, *SD* standard deviation

### Analysis

Qualitative data from the researcher reflection forms and the open-ended questionnaires were analysed through a process of descriptive coding [20]. Descriptive coding involves assigning labels to data that summarize in a word or a phrase the basic topic of a passage of qualitative data and is helpful when working across data forms [20] (e.g. reflection forms and open-ended questionnaires). The data was first read once without coding. Then, the data was re-read and coded inductively by one researcher (MW). Once all the data was coded, codes were compared and re-grouped into descriptive themes. Three authors met to discuss coding (MP, MW and ES). Responses to the question “is it clear?” were compiled from the CFIR Card Game Recording Sheet in an Excel file counting the number of implementation teams that identified the construct barrier narrative as unclear.

## Results

Four themes describing researchers and implementation team members’ experiences and perceptions emerged from this analysis: (1) the CFIR Card Game as a useful and engaging process, (2) difficulties understanding CFIR construct barrier narratives, (3) strengths of the game’s design and structure and room for improvement and (4) mediating factors: facilitator preparation and multi-stakeholder dynamics. Quantitative findings regarding the clarity of the barrier narratives were integrated with qualitative data under the theme complexity and understandability of the CFIR construct barrier narratives. Implementation teams and sites (organizations) are identified by Canadian province. Researchers and implementation team members’ quotes are identified by stakeholder group and Canadian province.

### The CFIR Card Game as a useful and engaging process

Participants overall found the game engaging and useful. Data was coded to this theme from 11 researcher reports and 34 open-ended questionnaire responses across all sites. The terms *fun*, *interactive* and *dynamic* were repeatedly used to describe the experience of playing the game. The group aspect of the game, which enabled everyone to participate and learn other team members’ opinions and perspectives, was repeatedly mentioned when describing what they like about the game. The engaging nature of the game contributed to the game’s usefulness. Learning colleagues’ perspectives during the game helped open participants’ minds to other perspectives and gauge what page everyone was on. Not only was the process useful, but so were the results—that is the identification of potential implementation barriers. Participants commented on how the game guided them to think about challenges, narrow down, make decisions through consensus and plan ahead. In the words of a service provider: “This activity helped in the planning for our innovation. Identifying barriers + strengths is essential” (New Brunswick 1). One manager was especially enthusiastic: “Loved! Will use in future decision making” (Manitoba 1). Only one participant expressed an overall negative view of the game and wrote: “probably not needed. Just provide the information on paper” (Knowledge user, New Brunswick 2).

### Difficulties understanding CFIR construct barrier narratives

Overwhelmingly, participants enjoyed the game, but even so, the most common response to what people did not like about the game had to do with finding it complex and the wording hard to understand. Data was coded to this theme from 11 researcher reports and 15 open-ended questionnaire responses across all sites. Many found the material was hard to follow, the wording confusing or too detailed and therefore hard to read and understand. It was not only the vocabulary but the tenses that caused some confusion. As one participant noted, the present tense was used in the barrier narratives when the prompting question was about future challenges (Knowledge user, Ontario). Another participant noted that what could improve the game would be providing each person with a separate sheet with all the definitions (as opposed to just projecting them on the board and reading them out loud) (Manager, Ontario).

This feedback is consistent with the quantitative data (Table 3). Only seven of 39 barrier narratives were clear to all teams and included “Cost”, “Cosmopolitanism”, “Relative Priority”, “Available Resources”, “Individual Stage of Change”, “Individual Identification with the Organization” and “Executing”. Eleven barrier narratives were unclear to four or more teams (out of seven). These were “External Policy & Incentives” (seven teams), “Complexity” (six teams), “Champions” (six teams), “Key Stakeholders” (five teams), “Relative Advantage”, “Trialability”, “Patient Needs & Resources”, “Compatibility”, “Learning Climate”, “Opinion Leaders” and “External Change Agents” (the last eight constructs identified as unclear by four teams). The number of constructs considered unclear by site ranged from three to 27 (Table 3). The researcher whose site found the greatest number of barrier narratives unclear reflected in their researcher reflection form that “the “plain” language translation was a good idea” and that “The CFIR is very technical, so having the constructs in plainer language helped with the transfer of knowledge.” (Researcher, New Brunswick 1 and 2). One participant in Ontario commented on the open-ended questionnaire that what they liked about the game was that it “provided plain language interpretation” (Manager, Ontario).

**Table 3:** Responses to the question “is it clear?” for each CFIR construct barrier narrative by implementation team

CIFR constructs (see Additional file 1 for barrier narratives)	QC	ON	MB1	MB2	NB1	NB2	BC	Number of implementation teams who responded “No”
Intervention Source	Y	N	N	Y	Y	N	Y	3
Evidence Strength & Quality	Y	Y	Y	Y	Y	N	Y	1
Relative advantage	Y	N	N	Y	N	N	N	5
Adaptability	Y	N	Y	Y	Y	Y	Y	1
Trialability	Y	N	Y	Y	N	N	N	4
Complexity	N	N	N	Y	N	N	N	6
Design Quality and Packaging	Y	Y	Y	Y	Y	N	Y	1
Cost	Y	Y	Y	Y	Y	Y	Y	0
Patient Needs & Resources	Y	N	Y	Y	N	N	N	4
Cosmopolitanism	Y	Y	Y	Y	Y	Y	Y	0
Peer Pressure	Y	N	N	N	Y	Y	Y	3
External Policy & Incentives	N	N	N	N	N	N	N	7
Structural Characteristics	Y	N	Y	Y	N	N	Y	3
Networks & Communications	Y	Y	Y	Y	Y	N	N	2
Culture	Y	Y	Y	Y	Y	N	N	2
Implementation Climate	Y	Y	N	Y	Y	N	N	3
Tension for Change	Y	Y	Y	Y	N	N	N	3
Compatibility	Y	N	Y	N	N	N	Y	4
Relative Priority	Y	Y	Y	Y	Y	Y	Y	0
Organizational Incentives & Rewards	Y	N	Y	Y	Y	N	N	3
Goals and Feedback	Y	N	Y	Y	Y	N	Y	2
Learning Climate	Y	N	N	N	Y	N	Y	4
Readiness for Implementation	Y	N	Y	Y	Y	N	Y	2
Leadership Engagement	Y	Y	Y	Y	Y	N	N	2
Available Resources	Y	Y	Y	Y	Y	Y	Y	0
Access to knowledge and information	Y	Y	N	Y	N	N	Y	3
Knowledge & Beliefs about the Intervention	Y	Y	Y	Y	Y	N	Y	1
Self-efficacy	Y	Y	Y	Y	Y	N	Y	1
Individual Stage of Change	Y	Y	Y	Y	Y	Y	Y	0
Individual Identification with Organization	Y	Y	Y	Y	Y	Y	Y	0
Planning	Y	N	Y	Y	N	Y	Y	2
Opinion Leaders	N	N	Y	N	N	Y	Y	4
Formally appointed internal implementation leaders	Y	N	Y	Y	N	N	Y	3
Champions	Y	N	N	N	N	N	N	6
External Change Agents	Y	N	Y	N	N	N	Y	4
Key Stakeholders	Y	N	N	N	N	N	Y	5
Patients/Customers	Y	Y	N	Y	Y	Y	Y	1
Executing	Y	Y	Y	Y	Y	Y	Y	0
Reflecting & Evaluating	Y	Y	Y	N	N	N	Y	3
Total unclear barrier narratives per site	3	20	11	9	16	27	22	

*BC* British Columbia, *MB* Manitoba, *ON* Ontario, *QC* Québec, *NB* New Brunswick, *Y* yes, *N* no

### Strengths of the game’s design and structure and room for improvement

Under the previous headings, feedback and suggestions from participants regarding the clarity of the statements was provided. Here, we summarize other feedback gleaned from the data pertaining to other specific aspects of the design and structure of the game including general design, time, framework used and introductory video. Data coded to this theme came from 6 researcher reports and 9 open-ended questionnaire responses from 5 sites. Design aspects that were valued included that the game was visual, organized, enabled participation from everyone and went through every construct. Perspectives on time were split. Two participants explicitly wrote that what they did not like about the game was that it was too long. However, two other participants suggested that the game needed more time and more time for discussion. There was a positive side to it being fast-paced from the perspective of a service user on an implementation team and that was that this led to “quick decision – on the spot” (Québec). One researcher reflected that it was a good idea to split the game across two meetings as it would be impossible to cover all constructs in one. However, despite splitting the game across two meetings, a researcher at another site was unable to complete the inner setting domain in one meeting and this spilled over into the second meeting, thus squeezing time even further. An alternative format of a 1-day workshop rather than two separate meetings was suggested by a manager (New Brunswick 1).

Regarding the CFIR framework explicitly, one participant stated both a positive and a negative aspect, with the positive being that there was a framework, and the negative being that the framework felt too directive (Service provider, Québec). Another participant said that what could improve the game would be more background on understanding the origins of the CFIR list of constructs (Service provider, Québec). Finally, from the perspective of one researcher, prefacing the game with an introductory video [18] on implementation science made a positive impact:

I really enjoyed the video, and was happy to learn that the members liked it too, the service users in particular. One service user stated that it really helped her understand implementation. She felt that the analogy made sense and was able to apply it to what we are doing as an implementation team (Researcher, New Brunswick 1).

### Mediating factors: facilitator preparation and multi-stakeholder dynamics

The researcher reflection forms revealed two factors that mediated the experience of playing the game and its outcome on a given day: facilitator preparation and multi-stakeholder dynamics. This theme derived exclusively from the 11 researcher reports. In the researcher reflection forms, facilitators made reference to the importance of their own preparation for ensuring the game ran well: “I felt well prepared and of course it has a good/positive impact on the meeting I think” (Lead researcher, New Brunswick 2). The co-facilitator elaborated in their reflection forms about the preparations that had been made:

In terms of logistics, we’re really getting the hand of it. [name of co-presenter] and I prepared beforehand. We made cards for the CFIR games and glued them to coloured poster board so they would be sturdier. We prepared colorful poster boards for the answers as well. As always, we were there a bit early so had enough time to set up and chat with the members. (Researcher, New Brunswick 2)

Feeling confident about the objective of the meeting and gaining experience was also important. One researcher involved in two sites reflected that having the chance to repeat the exercise a second time with another group “makes me more confident and have a stronger grasp on our objective and the activities.” (Researcher, Manitoba 1 and 2). They linked feeling confident to being “clear on the activities and knew [knowing] what the trajectory of the meeting was.” (Researcher, Manitoba 1 and 2)

As mentioned earlier, difficulty understanding the terminology of the barrier narratives was a common experience. One researcher reflected how their own action of spending time introducing the game in clear terms helped get everyone “into the swing of things”:

There was some hesitation at first about having to do the game again and around understanding the Terminology. [name of Co-facilitator] and I addressed this by spending more time introducing the game in clear terms. We got into the swing of things… (Researcher, British Columbia)

These reflections highlight the importance of facilitator preparation and confidence for smooth execution of the game and how confidence builds with experience.

Finally, researchers also noted the impact that different stakeholder group presence or absence during the game had on the game itself and the way in which the CFIR itself emphasizes the knowledge of certain stakeholder groups over others. For example, “The staff really shined as they had the most knowledge about the inner setting.” (Researcher, New Brunswick 2). A similar sentiment was expressed by others:

Since we discussed the inner setting, staff played a more predominant role because they knew the organization best. Family and service users pitched in but it was more the manager that led the discussion. (Lead researcher, New Brunswick 1)

The staff seemed to take a bit of an “expert” role during this meeting. This was somewhat natural, as the CFIR constructs we were reviewing often had to do with the broader organizational perspective. (Researcher, Manitoba 2)

Despite the advantage staff had with their insider knowledge, the researcher reflected that the staff were not too dominating and that “the tenants (service users) did a fabulous job ensuring their voice was heard and speaking up.” (Researcher, Manitoba 2).

However, the focus on staff and managers to provide this insider knowledge caused discomfort in one instance, as described in the following:

The power dynamics were more equal, but the members seem to turn to [the manager] often. While this was once a source of clarity for the team, it now seems that this makes [the manager] feel like she has the final say on things and that the burden of the innovation will fall on their shoulders. (Researcher, New Brunswick 1]

A similar sentiment was noted among a tenant, who at one particular meeting found themselves to be the only tenant present: “[The service user] seemed tense, and made comments about being “in the hot seat” as the only present tenant.” (Researcher, Ontario).

Just as presence of certain perspectives shaped dynamics so did the absence of a particular stakeholder group: “The fact that the manager was not there for a big part of the meeting allowed for some other members (staff) to engage with more energy (Lead researcher, New Brunswick 2)”

## Discussion

Implementation theories, whether generalized theories, models or frameworks, are valuable for building the knowledge base (e.g. by using theories to frame data collection and analysis) and for advancing the science of implementation [21]. However, they are also valuable for the implementation planning process. In our study, implementation teams were established in each site to plan for the implementation of a new recovery-oriented innovation into their services. We established a planning process (12 meetings) that included the development of the CFIR Card Game, not primarily as an academic implementation research exercise, but as a practical implementation planning exercise. The CFIR Card Game was aimed at translating implementation science frameworks and principles to the planning work of the implementation teams. Identifying barriers and strategies is an important part of implementation planning [22]. Without explicit focus on planning for implementation challenges and devising ways of addressing these, there is a risk that planning becomes only about the nuts and bolts of the specific innovation—thus limiting planning to the “what” of implementation and missing the “how”. Our impression throughout this project has been that implementation teams most naturally get excited about planning the innovation (e.g. designing a curriculum, choosing a program, deciding on venues etc.), and an important role of the researchers as the external facilitators was to insist on the importance of planning the implementation process. While we felt strongly that implementation teams should be identifying potential challenges for themselves and designing strategies into their plans, the length and density of both CFIR and ERIC posed a real practical challenge for their introduction into the team’s work process. From our reading of the implementation science literature, the focus has been on narrowing the gap between evidence-based innovations and their implementation into practice, whereas to do that also requires narrowing the gap between academic implementation science and real-world implementation planning. It is imperative that decision makers, practitioners and multi-stakeholder implementation teams can understand and use implementation science theories, frameworks and tools for planning, not just evaluation, especially when they are the ones designing their implementation plans. It can be harder to engage stakeholders in planning for implementation than designing innovations, and so creative approaches are needed [23]. Our CFIR Card Game created a bridge between theory and practice by drawing on the new CFIR-ERIC Matching Tool v.1 to design a process that was dynamic and interactive.

External change agents, whether researchers or consultants, play an important role in supporting organizational change and translating implementation science concepts and practices into implementation planning at individual and organizational levels [22, 24]. Organizations may lack support and expertise for implementation [25], thus reinforcing the role external change agents can play. Alagoz et al.’s systematic review on the use of external change agents for promoting quality improvement and organizational change found that simply providing information and advising on what should be done and in-person education are generally insufficient to achieve change [24]. Their review findings suggest “that a multi-faceted implementation strategy featuring regular, tailored follow up via practice facilitation is most likely to promote successful organizational change.” (p.12) [24]. Glasgow et al. put it the following way “to succeed, interventions must be implemented with methods that engage the partners and multiple stakeholders and that treat their varied perspectives with consideration and respect.” [26] (p. 5). As external change agents, we did not only want to apply the CFIR as a framework for data-collection at the post-implementation stage as it has typically been used [2]. Like others, we wanted to engage stakeholders in the implementation process to optimize planning and implementation success [22, 27–30]. Like others, we established and worked with implementation teams [31–34]. The inclusion of service users and family members on the implementation teams took the recovery principle of promoting collaborative relationships between service users and service providers [35, 36] and applied it to implementation planning. “Embedding innovations requires people to work together to solve emergent problems” [37] (p. 3) and our goal was to get teams to work together and actively engage with the CFIR so that they anticipate problems and incorporate strategies into their plans. What members liked about the game was hearing and learning from each other but also coming down to decisions as a team. A challenge is the fact that the CFIR, through its emphasis on internal organizational settings, can prioritize staff perspectives. However, this did not seem to be a barrier to participation from service users. Power dynamics and their impact on decision-making around anticipated barriers require further study. Also, from the perspective of researchers, stakeholder presence and absence was a mediating factor that merits further attention.

Importantly, we wanted to treat team members’ perspectives with respect [26] and design a way for them to identify their own challenges without overwhelming them. The game, as a process, worked well for introducing each construct in the CFIR to the teams and having them identify barriers. The majority found it fun, useful and interesting as it allowed them to hear and understand each other’s perspectives and come to quick decision-making as a team. They understood the importance of identifying challenges early. The process for matching these to strategies was empowering as it reinforced the fact that they had the power to do something about their challenges. It was important that researchers, as the external facilitators, felt confident in their role facilitating the game. However, the most common thing participants did not like about the game was that they found the language used hard to understand, complex and confusing. This was mentioned by all stakeholder groups, including managers, service providers and service users.

Few studies using the CFIR have reflected on the clarity of the CFIR terminology [2]. Since we were going to use the CFIR-ERIC Matching Tool v.1 to match challenges to strategies, it made strategic sense to use the barrier narratives provided in the tool as the text for the game cards. In our opinion, the barrier narratives were already the product of a process for simplifying the CFIR construct definitions as provided in the germinal article by Damschroder and colleagues in 2009 [1]. We investigated implementation teams’ impressions of the clarity of the CFIR-ERIC Matching Tool v.1 barrier narratives. While there was a lot of variation between teams in terms of what they found unclear, overall only seven barrier narratives were clear to all teams. More than half of the teams found 11 out of the 39 narratives unclear. This finding supports our assumption that plain language versions are needed in order for the CFIR-ERIC Matching Tool v.1 to be used and understood. While the tool narrows down complex concepts into short illustrative statements, the meaning of the constructs remains challenging to convey to non-specialists. The plain language versions we developed are a start but require further investigation, especially in bilingual contexts. For example, it is possible that New Brunswick 2 identified more constructs as unclear because they were predominantly French-speaking and found the less technical French versions helpful. In the bilingual setting of Montreal hearing, the original barrier narratives read twice, once in English and once in French, may have improved comprehension of the original statements thus accounting for why they identified fewer as unclear. The importance of continued work to simplify implementation science terminology cannot be overemphasized. The language needs to be “clear enough to withstand knowledge translation” to avoid the possible “wedge” unclear terminology can drive between researchers, service providers, service users and policy developers [17]. The language must be accessible for practitioners and stakeholders involved in the implementation process [17]. As implementation researchers and external change agents, we still have much to strive for in this regard.

### Limitations

The findings confirm that we were not wrong in our assumption that teams may struggle with the language used in the barrier narratives and we know that the plain language alternatives were appreciated. However, we did not evaluate whether participants would rate our plain language versions as clear on their own. This is a topic of ongoing research. This research highlights that for the CFIR and the CFIR ERIC Matching Tool v.1 to be used by organizations planning for implementation, more work needs to be done to translate these to plainer, more straightforward language.

By using the CFIR-ERIC Matching Tool v.1 barrier narratives for the cards, we framed our game around challenges rather than facilitators. This could be seen to contradict the strengths-based approach in recovery-oriented services [38] which emphasizes the importance of focusing on strengths, not weaknesses, in individuals, services and systems. A subsequent version could include statements re-oriented as facilitators. It is possible that social desirability (downplaying challenges or concealing one’s difficulty understanding) was at play, but knowing how would require further study. We also did not analyse the possible influence facilitator characteristics had on the game.

Furthermore, we did not study how power dynamics shaped responses to the question “is it clear?” We drove implementation teams to come to a consensus, but we know from other research on service user involvement in guideline development that the process of coming to a consensus in multi-stakeholder groups may still prioritize professional experience over lived experience and affect service user confidence in themselves in group settings [39]. One researcher’s reflection that the absence of the manager led to staff being more vocal points to power dynamics between staff and managers, not just staff and service users. Such dynamics deserve explicit attention in future research.

The authors of the CFIR-ERIC Matching Tool v.1 [4] caution that it should be used carefully because of the overall heterogeneity of the data on which the tool is based. It is possible that our use of the tool in this way is premature and that the tool requires further testing and elaboration to see whether the strategies proposed are indeed the most appropriate. However, the urgent need for a way to link strategies and challenges in practice justified this use. Future iterations of our CFIR Card Game should use the most up-to-date version of the CFIR-ERIC Matching Tool. An additional limitation of our approach was the pragmatic decision for researchers to select three key strategies to work on in meeting 10 with teams. We did not include a question on team members’ perspectives on the relevance or feasibility of the strategies selected by researchers. Although we did not observe any obvious differences between the opinions from the first site compared to the subsequent 6 sites who completed the game with a slightly different structure, we did not do a sub-group analysis to investigate this thoroughly.

## Conclusions

To the best of our knowledge, we are the first to transform the Consolidate Framework for Implementation Research into a Card Game. We drew on the CFIR-ERIC Matching Tool v.1 to design the game which helped match the challenges identified by teams to strategies teams could consider including in their implementation plans. The CFIR Card Game shows great promise as a process for enhancing planning and translating implementation science concepts to non-academic audiences working in real-world implementation settings. More work is needed to make CFIR construct language more accessible.

## Supplementary Information

**Additional file 1.** CFIR-ERIC Matching Tool v.1 Barrier Narratives (originals and plain language versions)

## Data Availability

All data generated or analysed during this study are included in this published article [and its supplementary information files].

## Abbreviations

CFIR: Consolidated Framework for Implementation Research
ERIC: Expert Recommendations for Implementing Change
