# Supplementary material for: The CFIR Card Game: a new approach for working with implementation teams to identify challenges and strategies
Source: Implement Sci Commun. 2021 Jan 7;2:1. doi: 10.1186/s43058-020-00099-1 (PMC7791817; doi:10.1186/s43058-020-00099-1)
Supplement: Supplementary file 1 — Additional file 1. CFIR-ERIC Matching Tool v.1 Barrier Narratives (originals and plain language versions) [file 43058_2020_99_MOESM1_ESM.docx]

**ADDITIONAL FILE 1**

Original CIFR-ERIC Matching Tool v.1 Barrier Narratives and Revised Plain Language Versions

Originals Barrier Narratives: Waltz TJ, Powell BJ, Fernández ME, Abadie B, Damschroder LJ. Choosing implementation strategies to address contextual barriers: diversity in recommendations and future directions. Implementation Science. 2019;14(1):42.

Plain language versions: Piat, M., Wainwright, M., Sofouli, E., Albert, H., Casey, R., Rivest, M-P, Briand, C., Kasdorf, S., Labonté L., LeBlanc, S., O’Rourke, J.. The CFIR Card Game: A new approach for working with implementation teams to identify barriers and strategies. Implementation Science Communications.

| **CFIR Construct [and Domain]** | **CFIR-ERIC Matching Tool v1 Barrier Narratives**  **(Waltz et al. 2019)** | **Plain Language Versions of CFIR-ERIC Matching Tool v.1 Barrier Narratives (Piat et al.)** |
| --- | --- | --- |
| INTERVENTION SOURCE  [characteristics of the intervention] | Stakeholders have a negative perception of the innovation because of the entity that developed it and/or where it was developed. | Stakeholders have a negative view of the innovation because of who developed it or because of where it was developed. |
| EVIDENCE STRENGTH & QUALITY  [characteristics of the intervention] | Stakeholders have a negative perception of the quality and validity of evidence supporting the intervention. | Stakeholders have a negative view of the research supporting the innovation. They don’t think the research is of good quality or believable. |
| RELATIVE ADVANTAGE  [characteristics of the intervention] | Stakeholders do not see the advantage of implementing the innovation compared to an alternative solution or keeping things the same. | Stakeholders do not see why implementing this innovation is better than other available options, or just keeping things as they are. |
| ADAPTABILITY  [characteristics of the intervention] | Stakeholders do not believe that the innovation can be sufficiently adapted, tailored, or re-invented to meet local needs. | Stakeholders don’t think the innovation can be adapted enough to meet their local needs. |
| TRIALABILITY  [characteristics of the intervention] | Stakeholders believe they cannot test the innovation on a smaller scale within the organization or undo implementation if needed. | Stakeholders believe it’s not possible to try-out the innovation before fully adopting it, or undo the change if needed. |
| COMPLEXITY  [characteristics of the intervention] | Stakeholders believe that the innovation is complex based on their perception of duration, scope, radicalness, disruptiveness, centrality, and/or intricacy and number of steps needed to implement. | Stakeholders believe the innovation is complicated because they see it as involving lots of steps, taking a long time, and being disruptive. |
| DESIGN QUALITY AND PACKAGING  [characteristics of the intervention] | Stakeholders believe the innovation is poor quality based on the way it is bundled, presented, and/or assembled. | Stakeholders believe the innovation isn’t very good because of the way it has been packaged and presented. |
| COST  [characteristics of the intervention] | Stakeholders believe the innovation costs and/or the costs to implement (including investment, supply, and opportunity costs) are too high. | Stakeholders believe the cost of the innovation and the costs of implementing it are too high. |
| PATIENT NEEDS & RESOURCES  [Outer Setting] | Patient needs, including barriers and facilitators to meet those needs, are not accurately known and/or this information is not a high priority for the organization. | The organization doesn’t know what its clients needs are or what the challenges and opportunities are for meeting those needs. Finding out isn’t a big priority for the organization. |
| COSMOPOLITANISM  [Outer Setting] | The organization is not well networked with external organizations. | The organization is not well linked or networked with other organizations. |
| PEER PRESSURE  [Outer Setting] | There is little pressure to implement the innovation because other key peer or competing organizations have not already implemented the innovation nor is the organization doing this in a bid for a competitive edge. | There isn’t a lot of pressure or competition to implement the innovation because other similar organizations haven’t implemented it either. |
| EXTERNAL POLICY & INCENTIVES  [Outer Setting] | External policies, regulations (governmental or other central entity), mandates, recommendations or guidelines, pay-for-performance, collaborative, or public or benchmark reporting do not exist or they undermine efforts to implement the innovation. | There are no policies, regulations, mandates or guidelines from outside the organization (e.g. from government or large association) that support implementation. Or, the policies, regulations, mandates or guidelines that exist outside the organization get in the way of implementing the innovation. |
| STRUCTURAL CHARACTERISTICS  [Inner Setting] | The social architecture, age, maturity, and size of an organization hinders implementation. | The way the organization is structured (e.g. how big it is, how many departments it has, staff turnover, ratio of managers to total employees) makes implementation more difficult. |
| NETWORKS & COMMUNICATIONS  [Inner Setting] | The organization has poor quality or non-productive social networks and/or ineffective formal and informal communications. | The organization doesn’t have good communication or networks between employees or between different departments. |
| CULTURE  [Inner Setting] | Cultural norms, values, and basic assumptions of the organization hinder implementation. | The culture of the organization (the ways things are done, the values, and the way of thinking) makes implementation more difficult. |
| IMPLEMENTATION CLIMATE  [Inner Setting] | There is little capacity for change, low receptivity, and no expectation that use of the innovation will be rewarded, supported, or expected. | The organization doesn’t have what it takes to change, isn’t that willing to, and people don’t expect their use of the innovation to be rewarded, supported or expected. |
| TENSION FOR CHANGE  [Inner Setting] | Stakeholders do not see the current situation as intolerable or do not believe they need to implement the innovation. | Stakeholders do not think the service is in need of change or don’t think the innovation is needed. |
| COMPATIBILITY  [Inner Setting] | The innovation does not fit well with existing workflows nor with the meaning and values attached to the innovation, nor does it align well with stakeholders' own needs and/or it heightens risk for stakeholders. | There isn’t a good fit between the innovation and the existing workflows, systems, norms and values of the organization. |
| RELATIVE PRIORITY  [Inner Setting] | Stakeholders perceive that implementation of the innovation takes a backseat to other initiatives or activities. | Stakeholders feel that implementing the innovation isn’t given much importance because other initiatives or activities are a higher priority. |
| ORGANIZATIONAL INCENTIVES & REWARDS  [Inner Setting] | There are no tangible (e.g., goal-sharing awards, performance reviews, promotions, salary raises) or less tangible (e.g., increased stature or respect) incentives in place for implementing the innovation. | There are no rewards or incentives in place to encourage people to implement the innovation (ex: performance reviews, promotions, salary raises, increased status or respect). |
| GOALS AND FEEDBACK  [Inner Setting] | Goals are not clearly communicated or acted upon, nor do stakeholders receive feedback that is aligned with goals. | Stakeholders in the organization don’t receive clear information about organizational goals and don’t get feedback that relates to goals. |
| LEARNING CLIMATE  [Inner Setting] | The organization has a climate where: a) leaders do not express their own fallibility or need for stakeholders’ assistance or input; b) stakeholders do not feel that they are essential, valued, and knowledgeable partners in the implementation process; c) stakeholders do not feel psychologically safe to try new methods; and d) there is not sufficient time and space for reflective thinking or evaluation. | Leaders in the organization (executives, middle management, supervisors, team leaders) don’t show their weaknesses or ask for help from stakeholders. Stakeholders don’t feel valued or knowledgeable, don’t feel safe and supported to try new things, and don’t feel they have enough time and space to think about and evaluate their practice. |
| READINESS FOR IMPLEMENTATION  [Inner Setting] | There are few tangible and immediate indicators of organizational readiness and commitment to implement the innovation. | It’s not clear that the organization is ready and committed to implement the innovation. |
| LEADERSHIP ENGAGEMENT  [Inner Setting] | Key organizational leaders or managers do not exhibit commitment and are not involved, nor are they held accountable for implementation of the innovation. | Key leaders or managers in the organization are not involved in, committed to, or held responsible for implementing the innovation. |
| AVAILABLE RESOURCES  [Inner Setting] | Resources (e.g., money, physical space, dedicated time) are insufficient to support implementation of the innovation. | There aren’t enough resources (money, training, education, physical space, time) to support implementation of the innovation. |
| ACCESS TO KNOWLEDGE AND INFORMATION  [Inner Setting] | Stakeholders do not have adequate access to digestible information and knowledge about the innovation nor how to incorporate it into work tasks. | Stakeholders don’t have good enough access to clear information about the innovation or how to put it into practice in their work. |
| KNOWLEDGE & BELIEFS ABOUT THE INTERVENTION  [Characteristics of Individuals] | Stakeholders have negative attitudes toward the innovation, they place low value on implementing the innovation, and/or they are not familiar with facts, truths, and principles about the innovation. | Stakeholders don’t like the innovation, don’t understand it, and don’t put a lot of importance on implementing it. |
| SELF-EFFICACY  [Characteristics of Individuals] | Stakeholders do not have confidence in their capabilities to execute courses of action to achieve implementation goals. | Stakeholders aren’t confident they can do what’s needed to reach implementation goals. |
| INDIVIDUAL STAGE OF CHANGE  [Characteristics of Individuals] | Stakeholders are not skilled or enthusiastic about using the innovation in a sustained way. | Stakeholders don’t have the skills or aren’t that excited about continuing to use the innovation. |
| INDIVIDUAL IDENTIFICATION WITH ORGANIZATION  [Characteristics of Individuals] | Stakeholders' are not satisfied with and have a low level of commitment to their organization. | Stakeholders aren’t satisfied and aren’t really committed to the organization. |
| PLANNING  [Process] | A scheme or sequence of tasks necessary to implement the intervention has not been developed or the quality is poor. | A plan for implementing the innovation, including all the steps needed, hasn’t been made or is poorly done. |
| OPINION LEADERS  [Process] | Opinion leaders (individuals who have formal or informal influence on the attitudes and beliefs of their colleagues with respect to implementing the intervention) are not involved or supportive. | Individuals who can influence other peoples’ attitudes or beliefs are not involved or supportive. |
| FORMALLY APPOINTED INTERNAL IMPLEMENTATION LEADERS  [Process] | A skilled implementation leader (coordinator, project manager or team leader), with responsibility to lead implementation of the innovation, has not been formally appointed or recognized within the organization. | A person responsible for leading implementation in the organization has not been chosen or recognized. |
| CHAMPIONS  [Process] | Individuals acting as champions who support, market, or ‘drive through’ implementation in a way that helps to overcome indifference or resistance by key stakeholders are not involved or supportive. | Individuals who act as champions for the innovation by supporting, marketing, driving, or overcoming any indifference or resistance from key stakeholders, are not involved or supportive. |
| EXTERNAL CHANGE AGENTS  [Process] | Individuals from an outside entity formally facilitating decisions to help move implementation forward are not involved or supportive. | Individuals from an outside organization who help with decision-making and moving things forward, are not involved or supportive. |
| KEY STAKEHOLDERS  [Process] | Multi-faceted strategies to attract and involve key stakeholders in implementing or using the innovation (e.g., through social marketing, education, role modeling, training) are ineffective or non-existent. | Ways of attracting and involving key stakeholders in implementing the innovation (e.g. posters, pamphlets, information sessions, training, role modelling) have not been developed or don’t work. |
| PATIENTS/CUSTOMERS  [Process] | Multi-faceted strategies to attract and involve patients/customers in implementing or using the innovation (e.g., through social marketing, education, role modeling, training) are ineffective or non-existent. | Ways of attracting and involving clients in implementing the innovation (e.g. posters, pamphlets, information sessions, training, role modelling) have not been developed or don’t work. |
| EXECUTING  [Process] | Implementation activities are not being done according to plan. | Implementation activities are not being done as planned. |
| REFLECTING & EVALUATING  [Process] | There is little or no quantitative and qualitative feedback about the progress and quality of implementation nor regular personal and team debriefing about progress and experience. | There is little or no feedback about how implementation is going and no regular meetings to talk about progress or peoples’ experiences with implementation. |
